# Contrastive explanations for machine learning predictions in chemistry

**DOI:** 10.1186/s13321-025-01100-6

**Published:** 2025-09-23

**Authors:** Alec Lamens, Jürgen Bajorath

**Affiliations:** 1 Department of Life Science Informatics and Data Science, B-IT, LIMES Program Unit Chemical Biology and Medicinal Chemistry, University of Bonn, Friedrich-Hirzebruch-Allee 5/6, 53115 Bonn, Germany; 2 Lamarr Institute for Machine Learning and Artificial Intelligence, University of Bonn, Friedrich-Hirzebruch-Allee 5/6, 53115 Bonn, Germany

**Keywords:** Explainable artificial intelligence, Human reasoning, Contrastive explanations, Molecular features, Analogue comparison, Selectivity prediction

## Abstract

The concept of contrastive explanations originating from human reasoning is used in explainable artificial intelligence. In machine learning, contrastive explanations relate alternative prediction outcomes to each other involving the identification of features leading to opposing model decisions. We introduce a methodological framework for deriving contrastive explanations for machine learning models in chemistry to systematically generate intuitive explanations of predictions in high-dimensional feature spaces. The molecular contrastive explanations (MolCE) methodology explores alternative model decisions by generating virtual analogues of test compounds through replacements of molecular building blocks and quantifies the degree of “contrastive shifts” resulting from changes in model probability distributions. In a proof-of-concept study, MolCE was applied to explain selectivity predictions of ligands of D2-like dopamine receptor isoforms.

## Scientific contribution

We introduce the first approach for generating contrastive explanations of machine learning models in chemistry. The methodology generates explanations of predictions that are readily accessible, chemically intuitive, and interpretable at the molecular level of detail.

## Introduction

With the rise of artificial intelligence (AI), machine learning (ML) is increasingly applied in different fields to address many different prediction tasks. Typically, contemporary ML models are “black boxes”, excluding the possibility to explain their decisions based on human considerations [1–3]. This general limitation poses a problem for the acceptance of ML predictions in interdisciplinary research areas such as drug discovery [4, 5]. Given the black box nature of deep ML models, it comes as no surprise that the field of explainable AI (XAI) is substantially gaining in attention [5–8]. In XAI, computational approaches are investigated to provide explanations of ML predictions, either as a part of the modeling processes or retroactively, which is more often the case. To this end, a multitude of concepts and methods have been proposed to provide insights into the inner workings of ML models [8, 9]. These approaches aim to provide explanations for solitary model decisions (local explanations) or strive for global explainability by generalizing internal process patterns as indicators of model behavior [9]. Computational explanations then provide a basis for model interpretation, human understanding, and causal reasoning [10]. Accordingly, explanation methods predominantly focus on accurate predictions, rather than weakly predictive models and their output.

XAI approaches for generating local explanations have received particular attention. For instance, feature attribution methods [9, 11], such as locally interpretable model-agnostic explanations (LIME) [12] or SHapley Additive exPlanations (SHAP) [13] quantify the individual contribution of each feature to a model decision. In chemistry, feature attribution analysis is usually combined with mapping of features onto molecular structures to help explain and interpret prediction outcomes [14]. Other XAI approaches adapted for ML in chemistry include, for instance, anchors, a rule-based methodology [15, 16], or counterfactuals [17–19]. Feature attribution and rule-based methods have in common that they attempt to explain a particular prediction without taking alternative prediction outcomes (such as different class labels) into account. In other words, these methods explore the question “why was prediction *P* obtained?”. Notably, humans often try to explain a given event by considering not only the event itself, but alternative outcomes instead [20]. For predictions, this leads to the question “why was prediction *P* obtained but not *Q*?” [20]. For instance, counterfactual thinking reflects the tendency of gaining insights into decisions by comparing different outcomes [17]. In molecular ML, counterfactuals are defined as closely related test compounds (analogues) yielding different predictions (class labels) [18, 19]. Moreover, the underlying question why prediction* P* and not *Q* was obtained is not only addressed by comparing alternative outcomes, but also by attempting to contrast potential origins of different outcomes, leading to contrastive explanations [21]. In this case, a given event (prediction) *P* is formally defined as the *fact* and the alternative event *Q* as the *foil* [21]. Contrasting these events aims to reduce cognitive load (with available and potentially contributing features) and increase clarity of ensuing explanations. In XAI, both of the related yet distinct concepts of counterfactuals and contrastive explanations are utilized [22, 23]. In human and artificial intelligence, contrastive explanations are especially sought after if a different event (prediction) than the observed one was anticipated [21, 23]. In ML, extending the comparison of closely related test instances yielding opposing predictions via counterfactuals [19, 22], contrastive explanations aim to capture features having the greatest impact on differentiating the *fact* from the *foil* [22–24]. Therefore, subsets of features responsible for distinct model decisions are determined.

In this work, we have adapted and modified the concept of contrastive explanations for molecular ML and applications in chemistry. A theoretical framework was established to generate chemically intuitive contrastive explanations that are interpretable at the level of molecular structure. In a proof-of-concept investigation, the molecular contrastive explanations (MolCE) approach was applied to analyze selectivity predictions for receptor ligands based on a new molecular test system.

## Methods

### Theoretical background

In ML, the principal goal of contrastive explanations is obtaining subsets of features present or absent in test instances that are minimally required for reaching opposing predictions [23–25]. This is referred to as the identification of pertinent positive or pertinent negative feature sets, representing minimal sets of features that must be present or absent to yield a particular model decision, respectively [23–25]. Contrastive explanations can be searched for by systematic reduction and re-assembly of feature subsets and determining the impact on predictions. Such explanations can also be generated by perturbing feature sets for test instances to obtain increasingly contrasting outcomes compared to an original prediction [26], that is, generating perturbed feature sets of *foils* increasingly departing from the *fact* [26]. Notably, this represents a continuum of increasing feature perturbations that may or may not invert a class label prediction [26].

Formally, a model $f$ assigning test instances $x$ to different classes is applied to predict an original instance $x^{*}$, obtaining output probability $p$ for $y^{*} = \arg \max f\left( {x^{*} } \right)$ representing the *fact* class. Then, the model is applied to predict instance $x^{\prime }$ obtained following feature perturbation, yielding output probability $q$ for a *foil* class selected from all available classes. The *contrastive behavior* $\delta^{contr}$ is calculated as the normalized shift in the probability distribution of the *foil* relative to the *fact*:

1 $${\updelta }_{p, q}^{contr} = \frac{{p_{{y^{*} }} }}{{p_{{y^{*} }} + p_{{y^{\prime } }} }} - \frac{{q_{{y^{*} }} }}{{q_{{y^{*} }} + q_{{y^{\prime } }} }}$$

The probability distributions $p$ and $q$ are derived from $f\left( {x^{*} } \right)$ and $f\left( {x^{\prime } } \right)$, respectively. The terms $\frac{{p_{{y^{*} }} }}{{p_{{y^{*} }} + p_{{y^{\prime } }} }}$ and $\frac{{q_{{y^{*} }} }}{{q_{{y^{*} }} + q_{{y^{\prime}}} }}$ represent the relative prediction probability of the *fact* class compared to the *foil* class, that is, for the original test instance and after perturbation of the feature set, respectively. The $\delta^{contr}$ values range from 1 and -1, with a value of 1 accounting for a complete probability shift from the *fact* to the *foil class* and a value of 0 indicating no shift. Thus, following feature perturbation, positive values reflect “contrastive shifts” towards the *foil* class and negative values contrastive shifts towards the *fact* class, corresponding to increasing probability of the original prediction.

### Molecular contrastive explanations

**Fig. 1:**
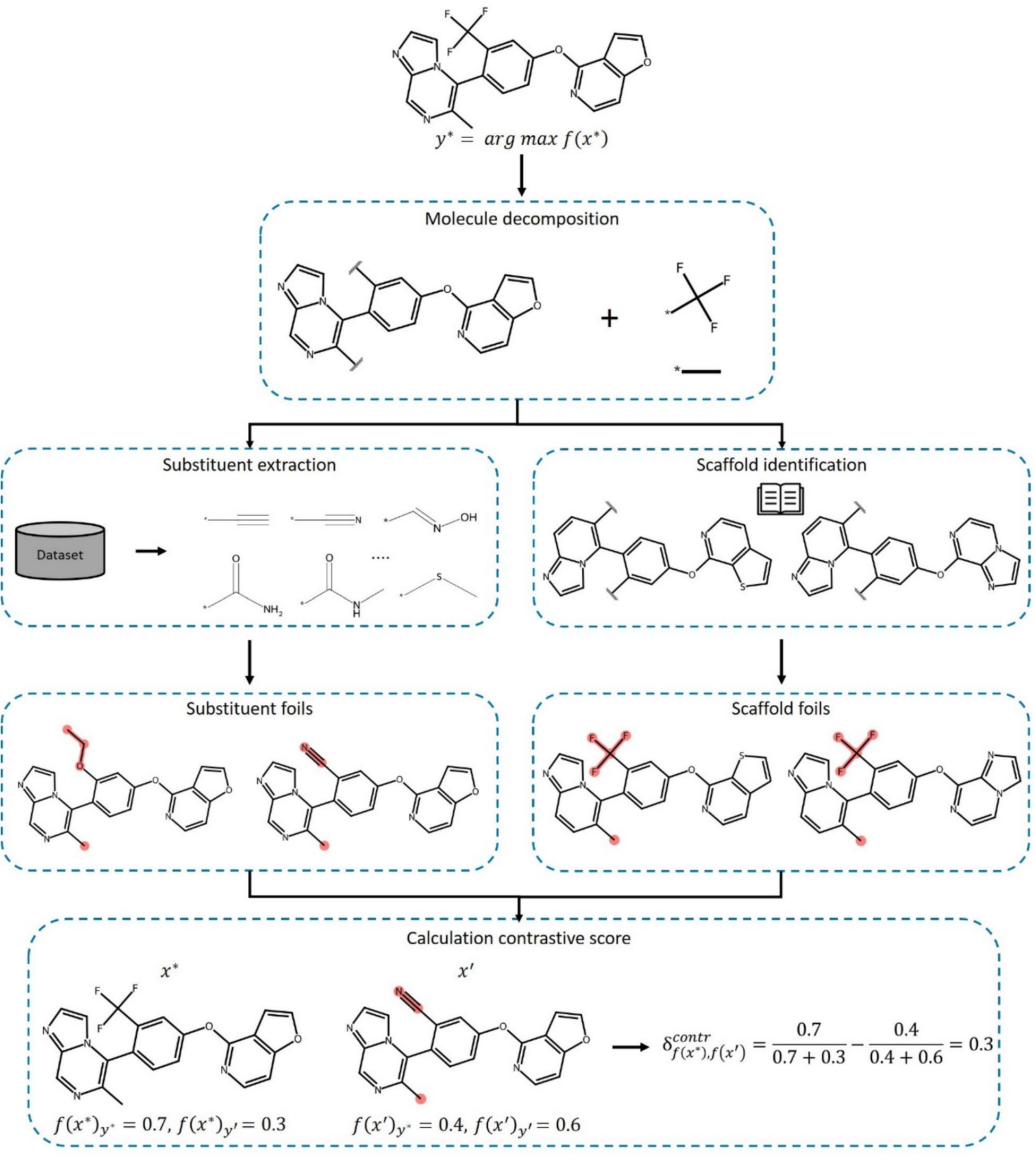
MolCE methodology. The application of the MolCE approach to an exemplary compound is summarized. Initially, the compound is decomposed into its scaffold and substituents. Alternative substituents are extracted from a reference data set and equivalent scaffolds are identified based on a reference dictionary of reduced carbon skeletons. Next, substituent foils and scaffold foils are generated through iterative substituent and scaffold replacement, respectively. Finally, the contrastive behavior is calculated for each foil

The MolCE approach adapts these principles for assessing contrastive shifts as a consequence of “molecular feature perturbation” resulting from the exchange of substituents or scaffolds (core structures) in given test instances, as illustrated in Fig. 1. Therefore, a test compound is decomposed into its substituents and scaffold according to Bemis and Murcko [27]. Then, virtual analogues are produced by replacing substituents or the scaffold (with a similar one, as detailed below). These virtual compounds represent *foils*. From a given data set used for ML, all unique substituents are extracted and iteratively added to the scaffold of a test instance at existing substitution sites, preserving one original substituent at a time while systematically replacing the others. For example, for a given scaffold with two substituents, each virtual analogue would include the original scaffold, one of the original substituents and a new substituent from the data set, producing a wealth of *substituent foils*. Additionally, virtual compounds are generated by combining the original substituents with selected scaffolds (see below), producing *scaffold foils*.

For this purpose, a reference dictionary was generated. For the creation of this dictionary, 1,115,950 unique compounds were extracted from BindingDB [28] and ChEMBL (version 35) [29], yielding a total of 658,659 unique scaffolds. These scaffolds were converted into carbon skeletons by replacing all heteroatoms with carbons and setting all bond orders to one. These carbon skeletons were further reduced (and generalized) by removing all linker atoms having two bonded neighbors. Accordingly, each reduced carbon skeleton represented a set of skeletons with varying linker lengths. The resulting 43,899 reduced carbon skeletons were stored in the dictionary that can be queried with a given reduced carbon skeleton extracted from a scaffold of interest to identify a set of alternative scaffolds. By design, these scaffolds are topologically closely related to the scaffold of interest.

For a test compound yielding a particular reduced skeleton, a variety of scaffolds were obtained. To ensure high similarity of original and alternative scaffolds, an atom-based size cut-off of 85% was applied, meaning that the number of atoms in alternative scaffolds could maximally differ 15% from the number of atoms in the original scaffold, thus focusing on very similar scaffolds. Then, in a test compound, the scaffold was systematically replaced with other qualifying candidates while retaining the original substituents.

The generation of *substituent foils* and *scaffold foils* ensured that feature perturbations led to chemically meaningful changes of test compounds corresponding to the applicability domain. Therefore *contrastive shifts* resulted from chemically relevant differences in molecular features (instead of artificial feature perturbations).

For a predicted test compound (*fact*), the most contrastive virtual analogues (*foils*) were identified by calculating the *contrastive behavior*, as detailed above. In the exemplary calculation at the bottom of Fig. 1, the model was applied to the original test instance ($x^{*}$) and virtual analogue ($x^{\prime }$) to obtain its prediction probabilities for the *fact class* ($y^{*}$) and *foil class* ($y^{\prime }$). The resulting probabilities were then used to calculate the *contrastive behavior* as defined in (1). Positive values indicate *contrastive shifts* of increasing magnitude.

The application of MolCE does not depend on specific classification or regression models and there is no intrinsic link between model performance and contrastive explanations of individual predictions.

### Selectivity data sets

For our proof-of-concept application of MolCE, compound selectivity data sets were generated. Selectivity for a given target over others is generally more difficult to predict than compound activity versus inactivity, and selectivity data sets are typically much smaller than compound activity classes (if they can be obtained at all). Thus, selectivity predictions represent a low-data application, which we deliberately selected for MolCE analysis.

Therefore, for our study, a compound test system for multi-class predictions comprising compounds selective for different D2-like dopamine receptors and non-selective compounds was assembled. Dopamine receptors are relevant pharmaceutical targets implicated in various neurological disorders such as Parkinson’s disease [30]. We selected the D2-like dopamine receptors family members D_2_R, D_3_R, and D_4_R, for which we could identify sufficient numbers of selective ligands for ML. While these receptor isoforms have high sequence similarity, their localization and function differ significantly [31]. For instance, D_2_R is primarily associated with antipsychotic drugs where common side effects include motor and endocrine irregularities [32]. Conversely, targeting D_4_R is a promising strategy to treat schizophrenia and pathologies resulting from opioid usage [32]. Hence, dopamine receptor isoform-selective compounds are sought after for the treatment of isoform-specific diseases [32, 33].

Receptor isoform pair-based selectivity data sets were generated using antagonists having pre-defined potency differences. A shared compound was classified as selective if the potency difference was at least 100-fold and as non-selective if the potency difference was at most tenfold. These criteria ensured that ligand selectivity was properly accounted for based on experimental potency differences.

Ligands of D_2_R, D_3_R, and D_4_R were extracted from BindingDB and CHEMBL, filtering out potential assay interference compounds and colloidal aggregators [34–36]. In addition, availability of a numerically specified potency value (IC_50_, K_i_, or K_d_) of at least 10 µM was required. Accordingly, compounds with activity in the low micromolar to low nanomolar range were selected. If a compound had multiple potency values falling into the same order of magnitude (that is, one log unit or less), the average of the values was calculated. By contrast, a compound was disregarded if the measurements differed by more than one log unit.

Two selectivity data sets were assembled including target pairs D_2_R–D_4_R and D_3_R–D_4_R. As expected, these data sets contained more non-selective (142–735) than selective compounds (18–230), as reported in Table 1. In addition, the numbers of unique analogue series (AS; compounds having the same core structure and different substituents) and singletons identified using the compound-core relationship algorithm are reported [37]. The distribution of AS and singletons relative to the total number of compounds reflected a comparable degree of chemical diversity across all selectivity classes.

**Table 1:** D2-like dopamine receptor selectivity data sets

Isoform pair	Label	# CPDs	# AS	# Singletons
D_2_R–D_4_R	Non-selective	735	101	86
	D_2_R-selective	29	6	5
	D_4_R-selective	230	25	10
D_3_R–D_4_R	Non-selective	142	25	42
	D_3_R-selective	18	4	3
	D_4_R-selective	37	5	6

### Balanced random forest models

To distinguish between selective and non-selective compounds, balanced random forest (BRF) models [38] controlling data imbalance were generated using scikit-learn [39]. For each prediction task, 10 independent trials were carried out by partitioning compounds into training sets (70%) and test sets (30%) using the “StratisfiedShuffleSplit” function of scikit-learn. BRF hyperparameters included the minimal number of samples for a leaf node (1, 2, 5, 10), minimal number of leaves required for a split (2, 3, 5, 10), and the number of decision trees (25, 50, 100, 200, 400). These hyperparameters were optimized using the training set partitioned into validation sets (70/30%) and GridSearchCV [39]. Compounds were represented using a folded extended-connectivity fingerprint with bond diameter 4 (ECFP4) [40].

### Performance measures

To assess the ability of models to distinguish between non-selective and selective ligands, several performance metrics were applied including balanced accuracy (BA) [41], precision, recall, F1 score (F1) [42] and Matthew’s correlation coefficient (MCC) [43].

$${\text{BA}} = { }\frac{1}{2}\left( {{\text{TPR}} + {\text{TNR}}} \right)$$

$${\text{Precision}} = \frac{TP}{{TP + FP}}$$

$${\text{Recall}} = \frac{TP}{{TP + FN}}$$

$$F1 = 2 \times \frac{TP}{{2TP + FP + FN}}$$

$${\text{MCC}} = { }\frac{{{\text{TP}} \times {\text{TN}} - {\text{FP}} \times {\text{FN}}}}{{\sqrt {\left( {{\text{TP}} + {\text{FP}}} \right)\left( {{\text{TP}} + {\text{FN}}} \right)\left( {{\text{TN}} + {\text{FP}}} \right)\left( {{\text{TN}} + {\text{FN}}} \right)} }}$$

TP abbreviates true positive predictions; FP false positives; TN, true negatives and FN, false negatives. TPR refers to the true positive rate and TNR to the true negative rate.

Macro-averaging was applied to the F1 score, precision, and recall. Accordingly, the metrics were calculated separately for each class and the arithmetic mean was determined as the final value.

## Results and discussion

### Classification performance

**Fig. 2:**
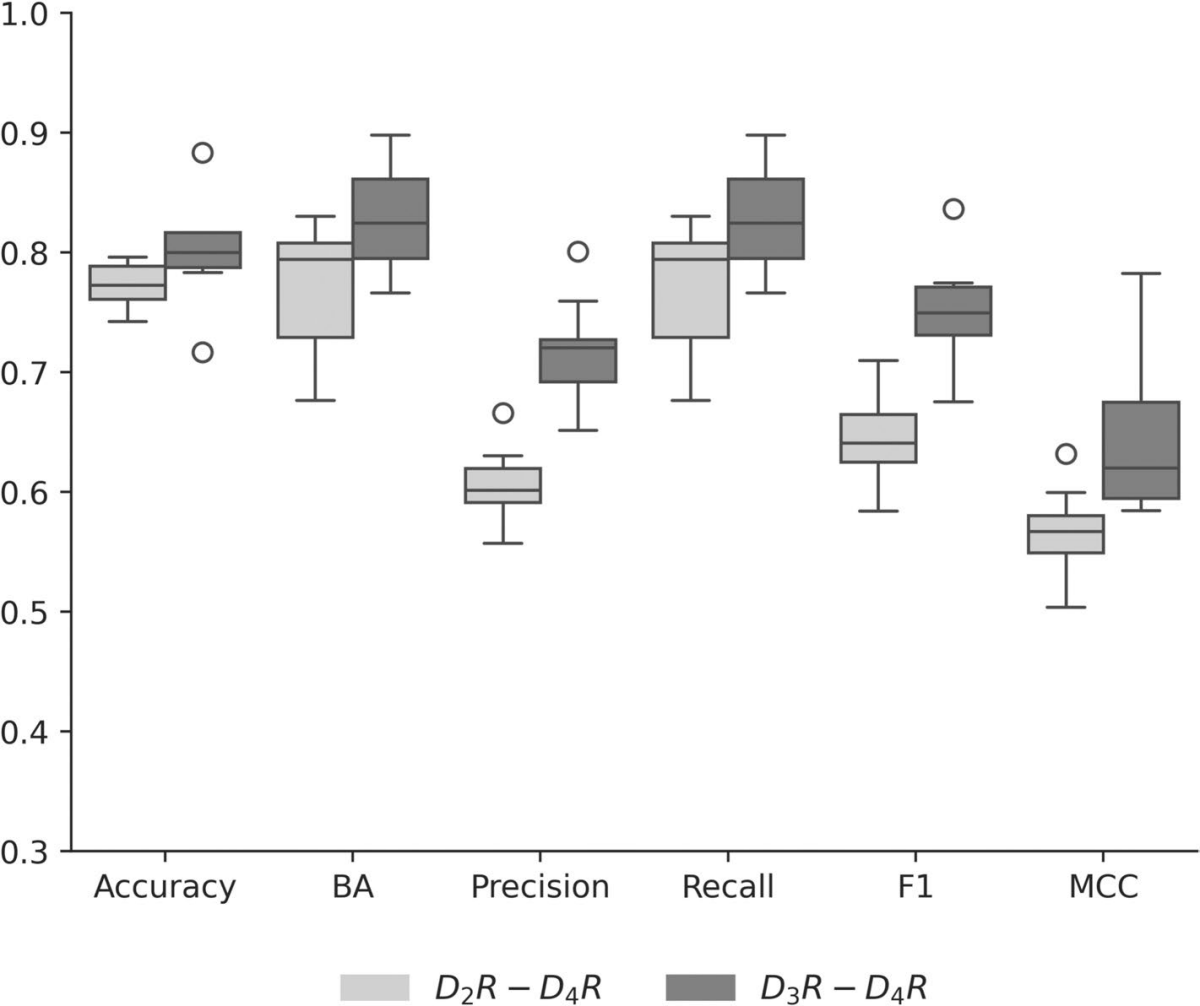
Model performance. Boxplots report the performance of BRF models on dopamine receptor selectivity data sets across 10 independent trials as based on different metrics

For both selectivity data sets, the ability of BRF models to distinguish between non-selective and selective compounds was determined. Figure 2 summarizes the performance of the BRF models across 10 independent trials.

BRF models adequately distinguished selective from non-selective compounds with a global accuracy of ~ 80%. Overall performance based on MCC values was marginally higher for the D_3_R–D_4_R compared to the D_2_R–D_4_R data set, with median values of 0.62 and 0.57, respectively. Precision values were consistently lower than recall, with median values of 0.60 and 0.72 for precision and 0.79 and 0.82 for recall, respectively. Thus, most positive instances were correctly predicted for the non-selective and selective classes. For comparison, other ML methods we tested including support vector machines with different kernels had lower performance for selective classes, with median balanced accuracy values ranging from 0.64 to 0.71.

**Fig. 3:**
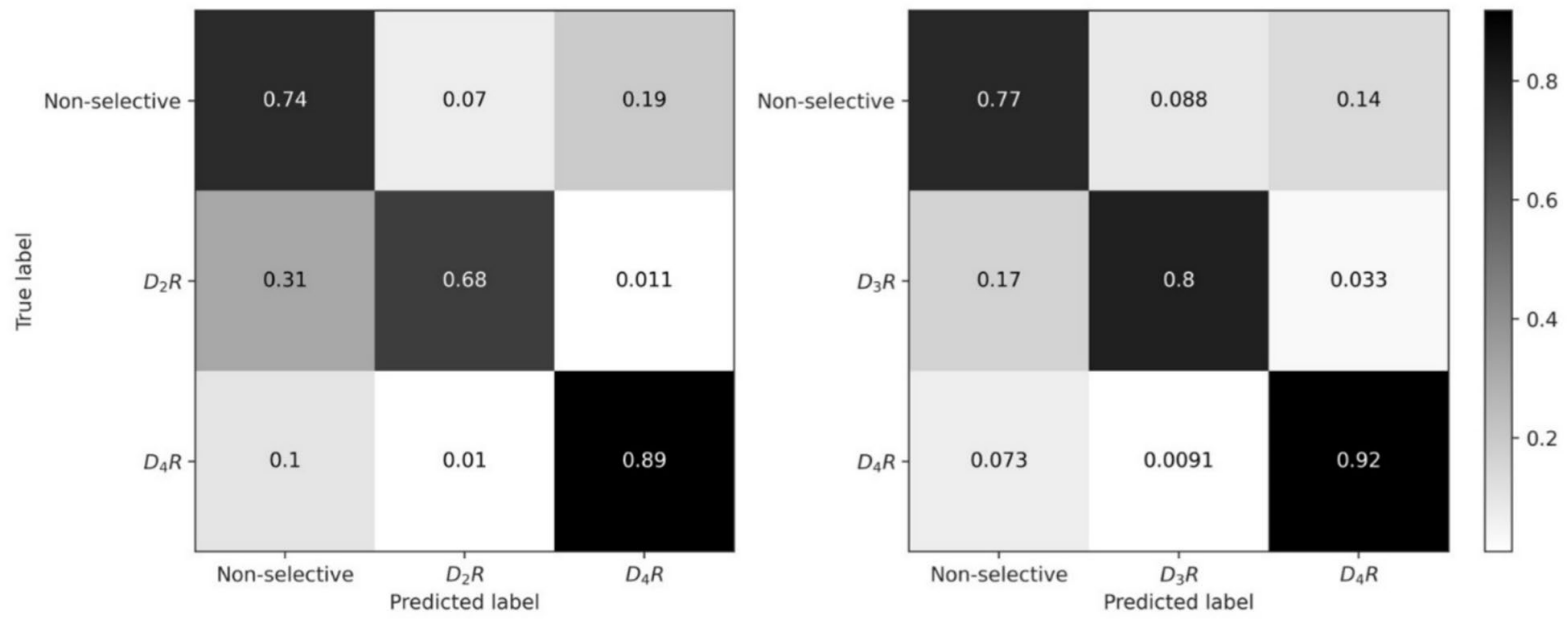
Class-dependent prediction accuracy. For the two selectivity data sets, confusion matrices report BRF model prediction accuracy over 10 independent trials. The matrices were normalized based on true labels

To further evaluate the performance of the BRF models, confusion matrices were generated, as shown in Fig. 3. The matrices revealed that the models had similar performance for selective and non-selective test compounds, with normalized accuracy values ranging from 0.68–0.92 to 0.74–0.77, respectively. Importantly, if selective compounds were incorrectly predicted, they were mostly predicted to be non-selective, rather than belonging to the other selectivity class. Similarly, for both selectivity data sets, incorrectly predicted non-selective compounds were mostly predicted to be D_4_R-selective. In this low-data application (see above), the overall high prediction accuracy for selective and non-selective test compounds provided a solid basis for subsequent MolCE analysis focusing on correctly predicted test compounds.

### MolCE statistics

**Fig. 4:**
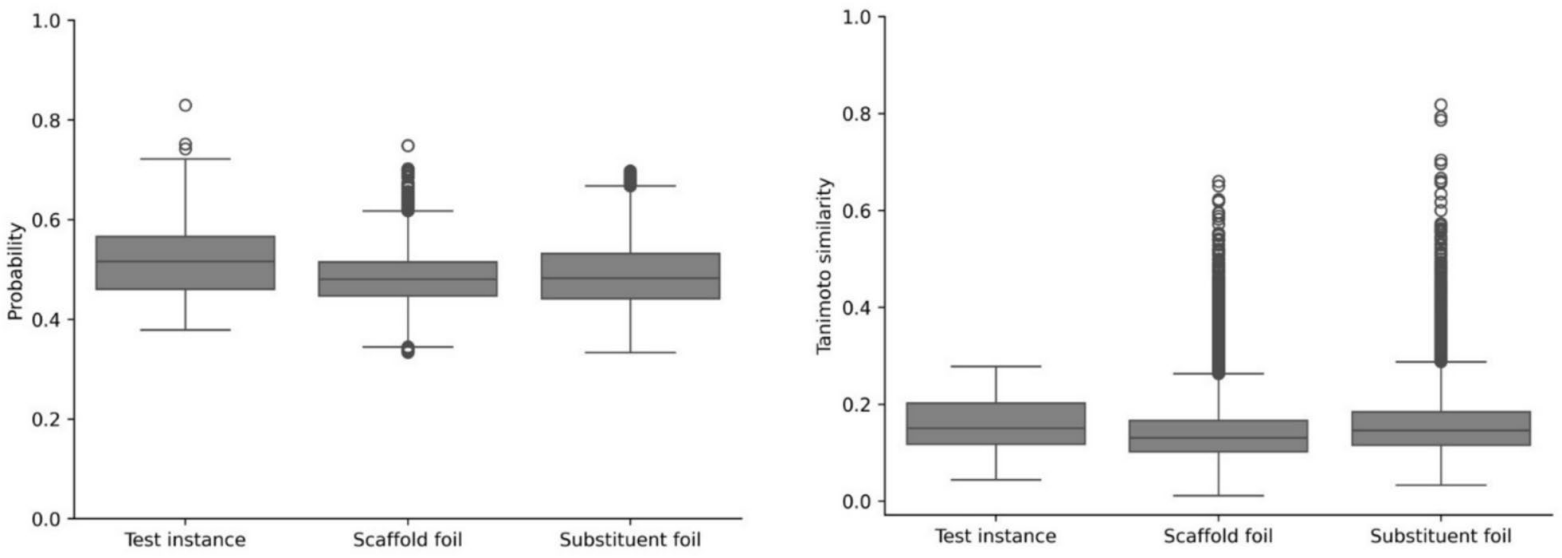
Applicability domain analysis. For an exemplary trial, all test set predictions and resulting scaffold and substituent foils were analyzed. Boxplots report the distribution of the highest class probability following model prediction of test instances or foils (left). Additionally, the distributions of pairwise Tanimoto similarity calculations for test instances or foils and all training instances are shown (right)

MolCE analysis was carried out for all correctly predicted non-selective and incorrectly predicted selective test instances. Correctly predicted non-selective compounds were of particular interest because determination of contrastive shifts enabled the identification of features driving the predictions of these compounds towards isoform selectivity. For this purpose, each set of selective compounds was considered a foil class. Conversely, for incorrectly predicted selective test instances, non-selective test compounds served as the foil class. This set-up enabled the identification of chemical features directing the incorrect prediction of non-selectivity (the fact) towards the correct prediction of selectivity (the foil). Additionally, it provided the basis for the analysis of chemical features determining correct predictions of compound selectivity that were not captured by incorrect predictions. First, it was established whether the contrastive examples fell into the application domain of the model. Figure 4 (left) shows the highest class probability following the prediction of test instances and generated scaffold or substituent foils. For BRF models, the maximal probability serves as a direct measure of model confidence. The probability distributions are similar for test instances and the generated foils, indicating a high degree of confidence in the test predictions and contrastive examples. Next, it was determined whether the generated contrastive examples were similar to the training compounds. Therefore, pairwise Tanimoto similarity calculations between scaffold and substituent foils and all training instances were carried out (Fig. 4, right). The similarity distributions of the contrastive examples are consistent with the distribution of the test instances, revealing that the contrastive examples fell into the application domain. Furthermore, to verify that contrastive shifts were not the result of large feature changes to compounds, the correlation between the contrastive shifts and Tanimoto similarity of the contrastive examples relative to test instances was determined. There was no detectable correlation for scaffold or substituent foils (with Pearson’s correlations ranging from -0.08 to -0.24). Thus, the observed contrastive shifts were the result of defined chemical changes and not cumulative feature perturbations.

**Fig. 5:**
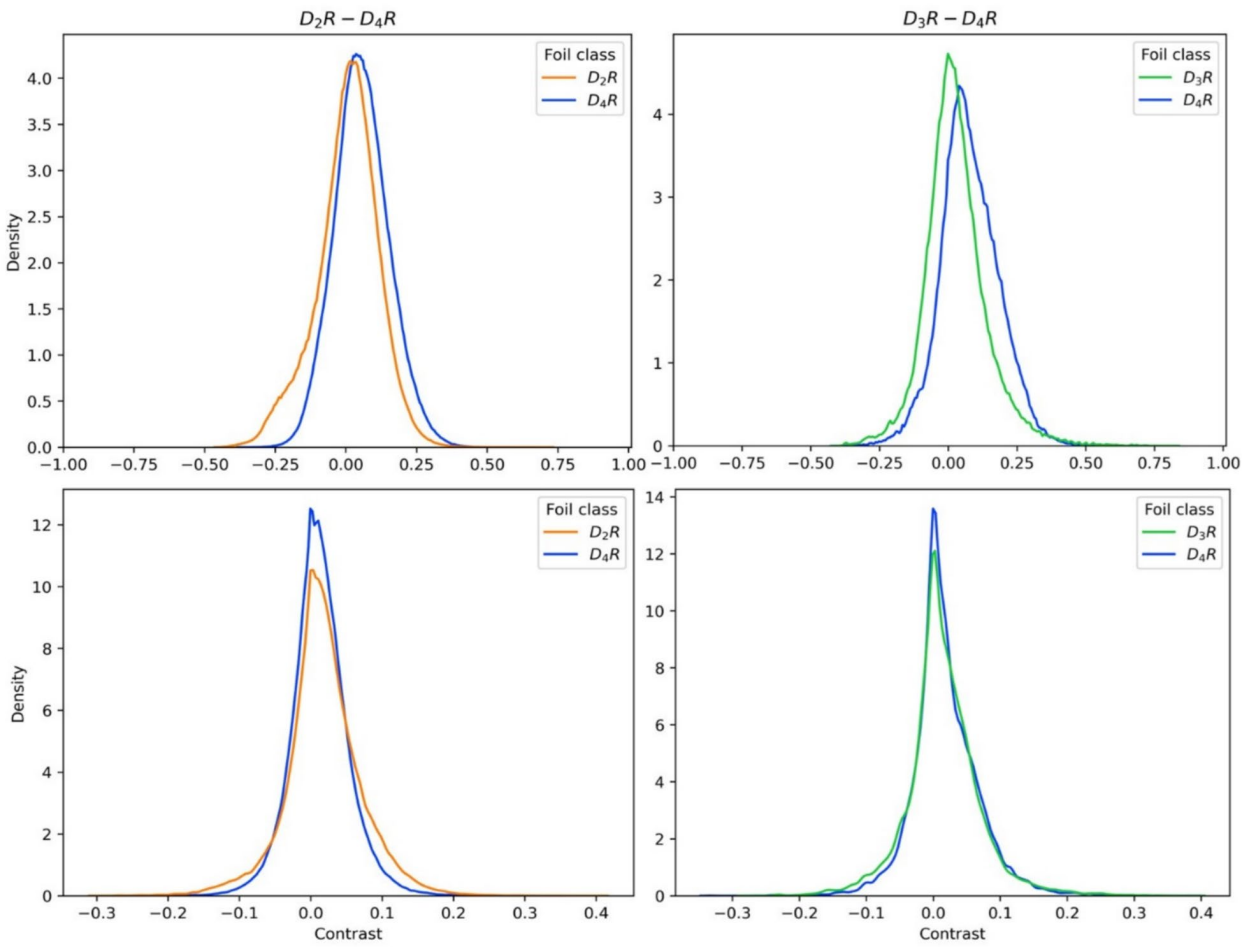
Contrastive behavior. Shown are the distributions of contrastive behavior values of all scaffold foils (top) and substituent foils (bottom) generated over 10 independent trials for data sets D2R–D4R (left) and D3R–D4R (right)

Figure 5 reports the contrastive behavior of all scaffold foils (top) and substituent foils (bottom) for D_2_R–D_4_R (left) and D_3_R–D_4_R (right) data sets, respectively. The distributions were similar for both selectivity data sets. Most scaffold foils showed only marginal contrastive behavior relative to the original prediction. This finding was attributable to the high similarity between the original and exchanged scaffolds. However, in each case, extreme values of the distributions identified a number of similar but highly-contrastive scaffold foils, with scores ranging from 0.46 to 0.83, indicating large contrastive shifts towards the *fact* or *foil class*. While corresponding observations were made for substituent foils, they generally caused contrastive shifts of smaller magnitude, with scores ranging from − 0.33 to 0.41.

Following our approach, the design of scaffold foils and substituent foils was structurally conservative, focusing on structural analogues, as it should be in order to arrive at chemically intuitive contrastive explanations. Importantly, the score distributions consistently included values indicating substantial contrastive shifts. Since sampling of our foils through scaffold or substituent replacements was computationally efficient, informative foils were consistently identified among large numbers of candidates. These scaffold foils and substituent foils captured small chemical modifications that disproportionately affected model decisions, revealing chemical features that were critically important for the ability of the BRF models to distinguish between fact and foil classes, as discussed in the following.

### Contrastive explanations

**Fig. 6:**
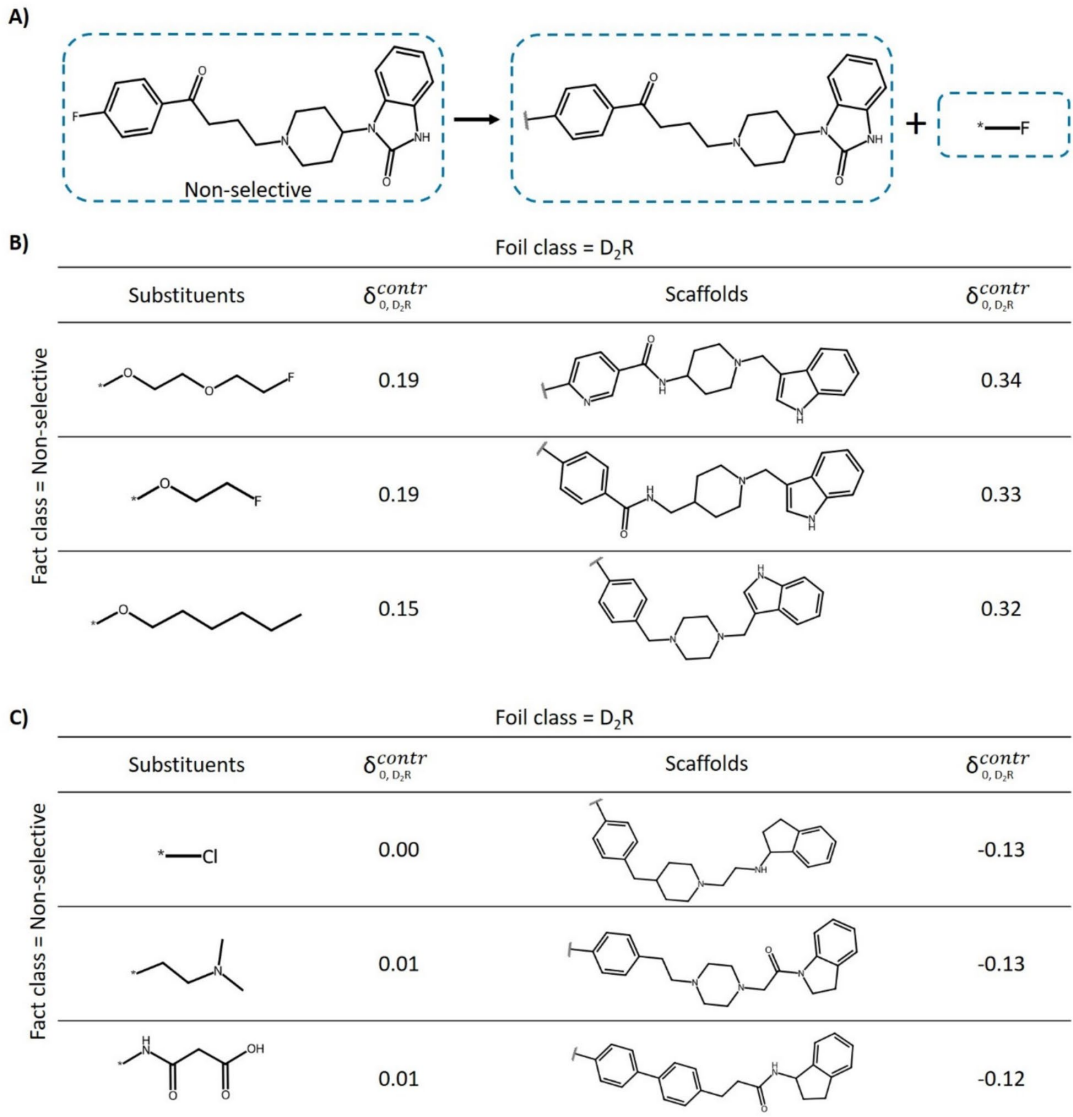
Contrastive explanation for a non-selective test compound. Shown is the application of MolCE to a correctly predicted non-selective test instance, with D_2_R-selective compounds representing *the foil class.* **A** The compound is decomposed into its scaffold and substituent. In **B** and **C**, the most and least contrastive substituents and scaffolds are shown, respectively

Figure 6a illustrates the application of MolCE to an exemplary correctly predicted non-selective test compound. In this case, D_2_R-selective compounds represented the foil class. Figure 6b shows the most contrastive alternative substituents and scaffolds that shifted the prediction towards D_2_R selectivity. Comparably large substituents containing ether moieties displayed the highest contrastive behavior. Moreover, small modifications of the original scaffold significantly impacted the prediction, with contrastive behavior scores ranging from 0.32 to 0.34. In the two most contrastive scaffolds, the original linker fragment was replaced with an amide linker. In all three contrastive scaffolds, the original imidazolidinone was replaced with a pyrroline moiety, revealing confined structural modifications that consistently caused contrastive shifts, indicating their relevance for the prediction of D_2_R selectivity. This observation was verified by determining the relative occurrence of the substructure across the data set. The pyrroline moiety was detected in 31% of the D_2_R-selective ligands compared to 9% and 10% of D_4_R- and non-selective ligands, respectively. The least contrastive substituents and scaffolds shown in Fig. 6c either had a negligible effect on the predictions or further shifted the probability in support of the fact class prediction. The original fluorine substituent was freely interchangeable with the least contrastive substituent, chlorine, as indicated by a score of 0. Furthermore, several similar scaffolds with negative scores were identified, thus further increasing the probability of the original prediction. Hence, on the one hand, different substituent and scaffold modifications were found to further support the correct prediction of non-selectivity; on the other, specific modifications were identified to cause substantial contrastive shifts towards the foil class.

**Fig. 7:**
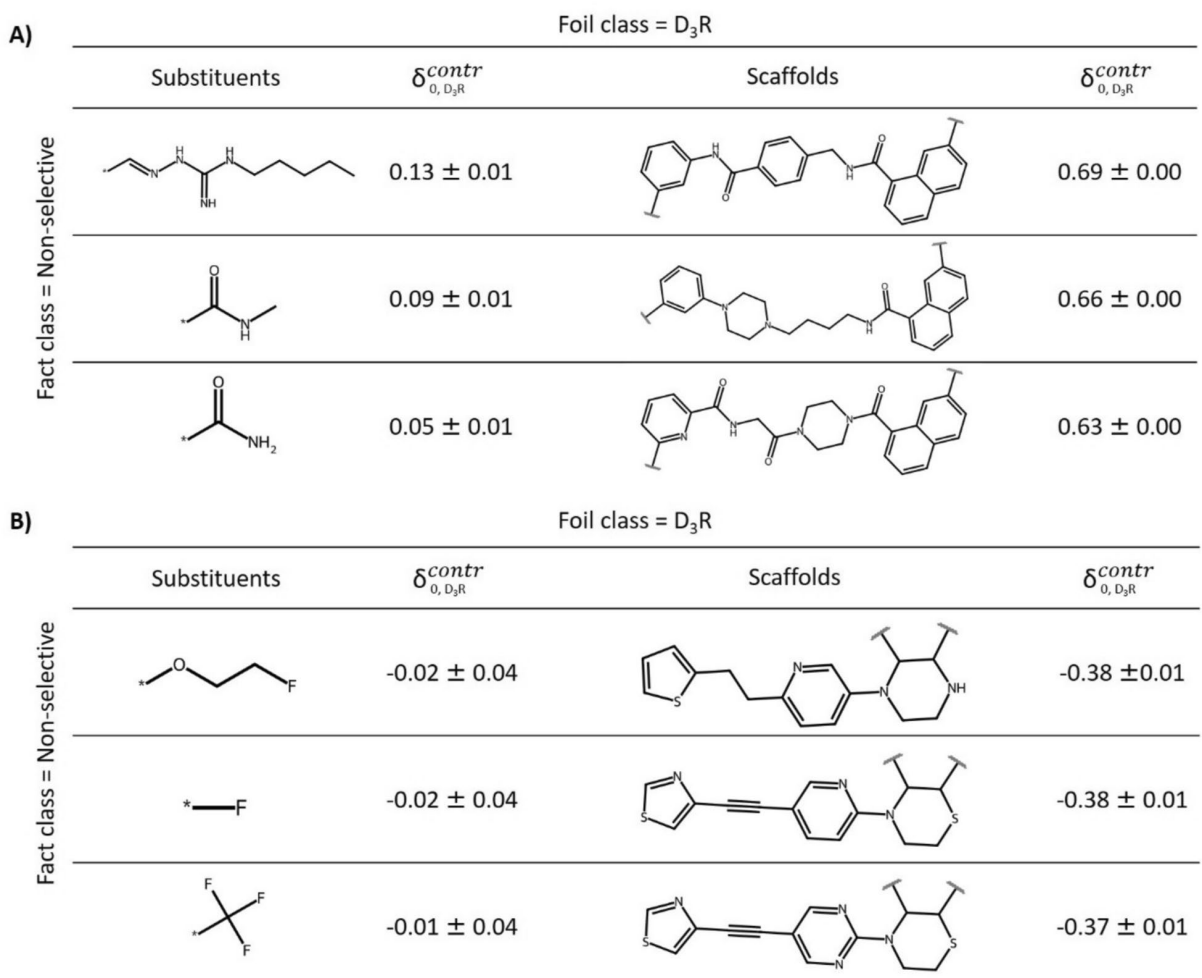
Global MolCE analysis of correct predictions. For all correctly predicted non-selective test compounds of the D_3_R–D_4_R data set across 10 independent trials, with D_3_R-selective compounds serving as the foil class, **A** and **B** show the most and least contrastive substituents and scaffolds, respectively, together with their mean contrastive behavior values

Next, we aggregated contrastive behavior for test compounds to search for global explanations. Therefore, contrastive behavior was calculated for all correctly predicted non-selective test compounds of the D_3_R–D_4_R data set over 10 independent trials, with D_3_R-selective compounds serving as the foil class. The values were averaged for all scaffolds from scaffold foils and substituents from substituent foils with single substitution sites to identify the most and least contrastive scaffolds and substituents, respectively (Fig. 7).

Figure 7a shows the most contrastive scaffolds and substituents. Mean contrastive shifts were much larger for scaffolds than substituents, indicating a key role of specific core structure modifications for distinguishing between non-selective and D_3_R-selective compounds. Highly-contrastive scaffolds contained amide linker and naphthalene moieties. In addition, consistently contrastive substituents were amine or amide derivatives. Conversely, Fig. 7b shows the least contrastive substituents and scaffolds, reinforcing correct predictions of non-selective compounds (with negative mean scores). These scaffolds contained heterocyclic amines combined with thiophene or thiazole moieties, which were not observed in highly-contrastive scaffolds. The least contrastive substituents contained fluorine substituents (including a single fluorine and trifluoromethyl group). Encouragingly, MolCE analysis identified global contrastive explanations for distinguishing between non-selective and D_3_R-selective test compounds. As an additional control, selectivity labels were randomized and the models re-trained using randomized data. For the resulting random predictions, the original contrastive shifts were no longer detectable.

**Fig. 8:**
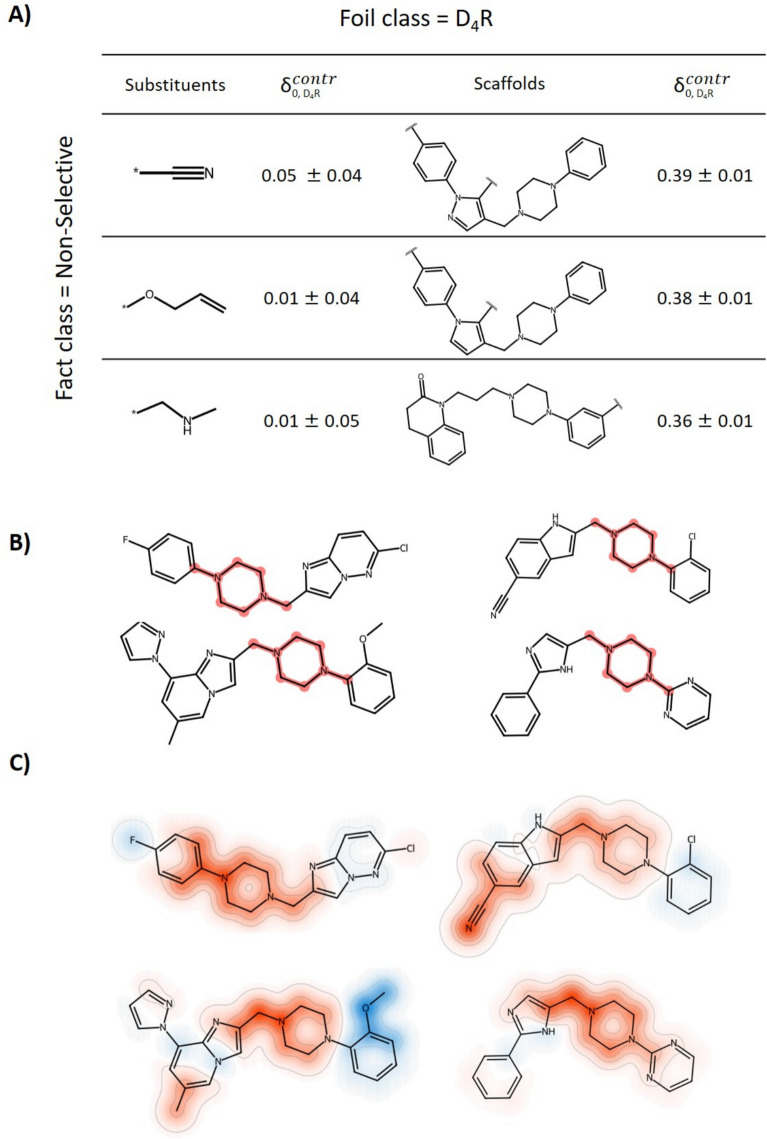
Global MolCE analysis of incorrect predictions. **A** For all D_4_R-selective test compounds from the D_2_R–D_4_R data set incorrectly predicted to be non-selective over 10 trials, with correctly predicted D_4_R-selective compounds serving as the foil class*,* the most contrastive substituents and scaffolds are shown. **B** Exemplary D_4_R-selective compounds containing the piperazine moiety (red) are shown. **C** For comparison, SHAP values were calculated with TreeExplainer [44] to quantify feature contributions. Values of features present in the D_4_R-selective test compounds were projected on their structures by atom-based mapping. Positive SHAP values (red) support the prediction of D_4_R-selectivity while negative values (blue) oppose this prediction

Furthermore, global MolCE analysis was carried out for all D_4_R-selective test compounds from the D_2_R–D_4_R data set that were incorrectly predicted to be non-selective, hence representing a prediction scenario different from the one discussed above. In this case, the fact class was the incorrect prediction of non-selectivity and the foil class the correct prediction of D_4_R selectivity. Accordingly, the most contrastive substituents and scaffolds shifted the probability distribution furthest towards the correct prediction outcome. As shown in Fig. 8a, contrastive substituents comprised carbonitrile, ether, and amine groups. Given their small positive scores, these substituents did not substantially affect the predictions. By contrast, contrastive shifts were much larger for scaffold foils. Notably, D_4_R-selective compounds contained different core structure variants, which was also reflected by the corresponding scaffold foils causing the largest contrastive shifts. The common feature of these scaffolds was the presence of a piperazine linked to a N-containing heterocyclic ring. In light of these observations, we determined the occurrence of the piperazine moieties in the core structures of the D_2_R–D_4_R data set. This substructure occurred in 65% of the D_4_R-selective ligands compared to 31% and 43% of D2R- and non-selective ligands, respectively. Figure 8b shows exemplary D_4_R-selective compounds containing a piperazine moiety. In all compounds, the piperazine substructure was also linked to a N-containing heterocyclic ring. These observations provided an intuitive explanation for the incorrect predictions, as the ML model preferentially associated D_4_R selectivity with this signature structural motif. However, the piperazine-heterocycle motif was not contained in all D_4_R-selective compounds including the incorrectly predicted instances. Figure 8c shows mapping of SHAP feature importance values on the D_4_R-selective compounds containing a piperazine moiety. On the basis of SHAP analysis, the piperazine motif also strongly contributed to the correct prediction of D4R-selectivity, consistent with the contrastive shifts for incorrect predictions of non-selectivity. However, SHAP analysis prioritizes large substructures for individual predictions, which is arguably more complex less clear than the focus on the piperazine motif considering contrastive shifts.

## Conclusions

The black box nature of contemporary ML models works against the acceptance of predictions for experimental design and thus limits the potential impact of ML in interdisciplinary research and development. Therefore, the field of XAI has steadily gained in attention over the past years. In general, XAI approaches aim to provide computational explanations for predictions or general insights into model behavior or internal processes. In the natural sciences, intuitive accessibility and interpretability of computational explanations play a critically important role for potential experimental follow-up. Due to their computational heterogeneity, not all XAI approaches are well suited for generating intuitive explanations of predictions that can be appreciated by an interdisciplinary audience. As a consequence, concepts originating from, for instance, psychology or the social sciences are sought after that can be adapted for XAI and have the potential to closely interface human and artificial intelligence. Among these is contrastive reasoning, reflecting a human tendency to explain non-anticipated events by concentrating on opposing outcomes. In XAI, contrastive explanations have thus far mostly focused on identifying feature subsets that are minimally required to ensure or prevent a particular prediction. In successful cases, this yields sound computational explanations for model decisions, but does not necessarily ensure interpretability and causal reasoning, especially in the natural sciences. We have devised the first adaptation of contrastive explanations for applications in chemistry, aiming to generate explanations that can be readily appreciated from a chemical perspective and further explored at the molecular level of detail. Therefore, the MolCE methodology generates virtual candidate molecules (instead of exploring ML feature sets) and quantifies their contrastive behavior compared to “factual” predictions. This makes it possible to determine contrastive shifts, select preferred candidate molecules, and analyze structural features contributing to contrastive explanations, as shown herein. The “fact-foil” framework for assessing contrastive behavior is versatile and capable of accounting for various prediction settings, making it possible to center the analysis in different ways. Intuitive access to and interpretability of predictions also plays a key role in medicinal chemistry-driven early-phase drug discovery. In support of candidate selection and compound synthesis, contrastive explanations focusing on compound structures and minimal changes are readily accessible to practicing chemists, without the need to consider theoretical foundations in detail. While complementary to feature attribution methods, contrastive explanations generated with MolCE often provide a clearer chemical picture of origins of prediction outcomes than quantification of feature contributions that is interpretable in chemical terms by comparing structural analogues. This is particularly attractive for introducing and evaluating compound predictions in the practice of medicinal chemistry. Taken together, the findings of our proof-of-concept study suggest that the MolCE approach will be of considerable interest for molecular ML and practical applications in chemistry. It is also noted that the approach should be applicable to further advance the analysis of molecular counterfactuals, for instance, by determining contrastive shifts as a consequence of minimal structural modifications, providing an opportunity for future research. Moreover, the method can be easily adapted to regression tasks by calculating contrastive shifts as a function of predicted values instead of class probabilities. As a part of our study, the new selectivity data sets and the MolCE method are made freely available.

## Data Availability

All data and code are available via the following link: https://uni-bonn.sciebo.de/s/UZmmShkogZrMbGQ.

## Abbreviations

AI: Artificial intelligence
AS: Analogues series
BA: Balanced accuracy
BRF: Balanced random forest
CPDS: Compounds
ECFP4: Extended-connectivity fingerprint with bond diameter 4
FN: False negative
FP: False positive
LIME: Locally interpretable model-agnostic explanations
MCC: Matthew’s correlation coefficient
ML: Machine learning
MolCE: Molecular contrastive explanations
SHAP: Shapley additive explanations
TN: True negative
TNR: True negative rate
TP: True positive
TPR: True positive rate
XAI: Explainable AI
